# Investigating the Role of *SNAI1* and *ZEB1* Expression in Prostate Cancer Progression and Immune Modulation of the Tumor Microenvironment

**DOI:** 10.3390/cancers16081480

**Published:** 2024-04-12

**Authors:** William Lautert-Dutra, Camila Morais Melo, Luiz Paulo Chaves, Francisco Cesar Sousa, Cheryl Crozier, Dan Dion, Filipe S. Avante, Fabiano Pinto Saggioro, Rodolfo Borges dos Reis, Leticia Fröhlich Archangelo, Jane Bayani, Jeremy A. Squire

**Affiliations:** 1 Department of Genetics, Faculty of Medicine at Ribeirão Preto, University of São Paulo (FMRP-USP), Ribeirão Preto 14049-900, SP, Brazil; williamlautert@alumni.usp.br (W.L.-D.); camilammelo@alumni.usp.br (C.M.M.); luizpaulocds@alumni.usp.br (L.P.C.); 2 Division of Urology, Department of Surgery and Anatomy, University of São Paulo (FMRP-USP), Ribeirão Preto 14049-900, SP, Brazil; franciscocesar@alumni.usp.br (F.C.S.); favante@hcrp.usp.br (F.S.A.); rodolforeis@fmrp.usp.br (R.B.d.R.); 3 Diagnostic Development, Ontario Institute for Cancer Research, Toronto, ON M5G 0A3, Canada; ccrozier@oicr.on.ca (C.C.); dan.dion@oicr.on.ca (D.D.); jane.bayani@oicr.on.ca (J.B.); 4 Department of Pathology, University of São Paulo (FMRP-USP), Ribeirão Preto 14049-900, SP, Brazil; fsaggioro@terra.com.br; 5 Department of Cellular and Molecular Biology and Pathogenic Bioagents, Ribeirão Preto Medical School, University of São Paulo (FMRP-USP), Ribeirão Preto 14049-900, SP, Brazil; leticiafa@fmrp.usp.br; 6 Laboratory Medicine and Pathology, University of Toronto, Toronto, ON M5G 1E2, Canada; 7 Department of Pathology and Molecular Medicine, Queen’s University, Kingston, ON K7L3N6, Canada

**Keywords:** biomarkers, immune evasion, bioinformatics, gene signatures, immunotherapy, extracellular matrix, transcriptomics, collagens, immune checkpoint proteins, digital flow cytometry

## Abstract

**Simple Summary:**

We evaluate the downstream effects of the Epithelial-to-Mesenchymal Transition (EMT) transcription factors, *ZEB1* and *SNAI1*, and analyze their potential significance as biomarkers for increased aggressiveness and immune response in prostate cancer (PCa). We used two commercial expression profiling panels to examine a primary PCa cohort (*n* = 51) and identified changes in gene expression linked to downstream pathways associated with biochemical recurrence and increased clinical risk. Genes such as *COL1A1*, *COL1A2*, and *COL3A1*, which are implicated in the tumor microenvironment, and immune-related genes, such as *THY1*, *IRF5*, and *HLA-DRA*, exhibited significant expression level changes. Enrichment analysis identified pathways associated with angiogenesis, TGF-beta, EMT, and UV response in PCa progression. Confirmatory analyses conducted using public domain data demonstrated the downstream impacts of *ZEB1* and *SNAI1* on pathways and immune responses, highlighting their potential influence on immune modulation in PCa. Future treatment strategies aimed at modulating EMT may enhance immune cell infiltration toward an anti-tumorigenic phenotype.

**Abstract:**

Prostate cancer (PCa) is an immunologically cold tumor and the molecular processes that underlie this behavior are poorly understood. In this study, we investigated a primary cohort of intermediate-risk PCa (*n* = 51) using two NanoString profiling panels designed to study cancer progression and immune response. We identified differentially expressed genes (DEGs) and pathways associated with biochemical recurrence (BCR) and clinical risk. Confirmatory analysis was performed using the TCGA-PRAD cohort. Noteworthy DEGs included collagens such as *COL1A1*, *COL1A2*, and *COL3A1*. Changes in the distribution of collagens may influence the immune activity in the tumor microenvironment (TME). In addition, immune-related DEGs such as *THY1*, *IRF5*, and *HLA-DRA* were also identified. Enrichment analysis highlighted pathways such as those associated with angiogenesis, TGF-beta, UV response, and EMT. Among the 39 significant DEGs, 11 (28%) were identified as EMT target genes for *ZEB1* using the Harmonizome database. Elevated *ZEB1* expression correlated with reduced BCR risk. Immune landscape analysis revealed that *ZEB1* was associated with increased immunosuppressive cell types in the TME, such as naïve B cells and M2 macrophages. Increased expression of both *ZEB1* and *SNAI1* was associated with elevated immune checkpoint expression. In the future, modulation of EMT could be beneficial for overcoming immunotherapy resistance in a cold tumor, such as PCa.

## 1. Introduction

Prostate cancer (PCa) is the second most common cancer in men and the fifth cause of cancer-related deaths worldwide [1,2]. The disease course is often favorable, but unfortunately, 20–30% of patients with localized disease will eventually progress and develop advanced disease and metastasis [3]. Once resistance to androgen deprivation therapy develops, there are limited chemotherapy choices available to control the progression [4], but recently there has been increasing interest in the use of immunotherapy in the advanced setting.

The effect of checkpoint blockade therapy in metastatic PCa has been disappointing, with just 5–10% of patients responding [5,6]. These poor results are primarily thought to be because PCa is an immunologically cold or excluded tumor [7,8]. In various solid tumors, the presence of immune infiltration within the tumor microenvironment (TME) has been associated with improved immune control and a better prognosis [9].

The TME is the cellular ecosystem that surrounds a tumor, and it includes immune cells, the extracellular matrix (ECM), blood vessels, and other cells, such as cancer-associated fibroblasts (CAFs) that may modulate the composition of the TME. Studies of the immune content in PCa have resulted in inconsistent findings, with some indicating that elevated T cell levels within the TME correlate with improved prognosis [10], while others suggest the opposite effect [11]. The variation in immune infiltration likely contributes to the observed differences in anti-cancer immune responses in PCa [12,13]. 

Epithelial–mesenchymal transition (EMT) mechanisms can profoundly influence the TME [14]. The EMT is a molecular mechanism associated with tumor progression and acquisition of heterogeneity in advanced cancers [15]. EMT-inducing transcriptional regulators, such as *TWIST*, *SNAI1*, *SNAI2*, *ZEB1*, and *ZEB2*, exert their phenotypic changes in tumors by modulating the expression of epithelial markers and activating the expression of mesenchymal markers [14]. These downstream regulatory changes in gene expression occur through their direct binding to the promoters of target genes involved in cell adhesion and polarity, leading to loss of cell–cell adhesion, remodeling of the cytoskeleton, and acquisition of migratory and invasive properties characteristic of mesenchymal cells [16].

Zinc finger E-box binding homeobox 1 (*ZEB1*) is an established EMT transcription factor whose expression in PCa is associated with more aggressive disease and chemoresistance [17]. Similarly, Snail family transcriptional repressor 1 (*SNAI1*) is the main promoter of EMT in PCa [18], and its expression is associated with a higher Gleason score [19] and increased cell migration [20].

EMT-driven alterations to the TME can lead to resistance to immunotherapy [21,22]. *TGF-β* signaling is integral to the epithelial phenotype and downstream effects induce changes in the stromal environment to facilitate tumor progression [23]. The expression of *TGF-β* interacts with both the Snail and ZEB1 proteins to influence cancer–TME crosstalk related to immune evasion [24,25,26].

The prognostic role of downstream EMT transcriptomics derived from PCa primary intermediate-risk tumors has not previously been investigated in the context of the immune landscape of the TME. In this study, we analyzed the influence of altered *ZEB1* and *SNAI1* expression levels on cancer progression using a retrospective cohort of 51 intermediate-risk PCa tumors from FMRP-USP, Brazil. We determined how downstream changes in gene expression related to each transcription factor could lead to PCa progression changes and immune pathway activities. We used two NanoString mRNA panels (PanCancer Pathway and Immune Profiling) to quantify gene expression levels across the cohort to identify differentially expressed genes (DEGs) and pathways linked to the EMT and progression in intermediate-risk PCa. Our findings indicate that changes in *ZEB1* and *SNAI1* expression in PCa are associated with the induction of DEGs and downstream pathways that influence the TME and may facilitate immune evasion during tumor progression.

## 2. Materials and Methods

### 2.1. Tumor Cohort

The Faculty of Medicine at the Ribeirão Preto (FMRP) cohort comprised 51 primary prostate cancer samples obtained via radical prostatectomy, in accordance with the National Comprehensive Cancer Network (NCCN) clinical practice guidelines [27], at the Urology Division of the Department of Surgery and Anatomy, FMRP-USP, Brazil, between 2007 and 2015 (Table S1). Transcriptomic data derived from this cohort were recently included in another publication by our group [28]. Smaller prostates were submitted for pathological assessment in their entirety according to the guidelines of the American College of Pathology. In cases where larger glands were partially sampled, we followed the protocol by submitting the entire tumor if grossly visible, along with the tumor, surrounding periprostatic tissue, and margins, including the entire apical and bladder neck margins. Additionally, we included the junction of each seminal vesicle with the prostate proper. If there was no grossly visible tumor, a systematic sampling strategy was used. This involved taking slices from the posterior aspect of each transverse section, along with a mid-anterior block from each side. Additionally, we submitted samples including the entire apical and bladder neck margins, as well as the junction of each seminal vesicle with the prostate. Biochemical recurrence (BCR) was defined as PSA > 0.2 ng/mL within six months post radical prostatectomy. To assess the likelihood of prostate cancer recurrence after initial surgery, we utilized the Cancer of The Prostate Risk Assessment Score (CAPRA-S) [29]. This scoring system incorporates various clinical and pathological factors, such as pre-treatment PSA level, pathological Gleason score, surgical margin, extracapsular extension, seminal vesicle invasion, and lymph node invasion. CAPRA-S provides a relative risk assessment for biochemical progression, ranging from 1 to 12. For this study, patients with low CAPRA-S scores were those with values between 0 and 2, those with intermediate scores had CAPRA-S scores ranging from 3 to 5, and those with high scores had CAPRA-S scores between 6 and 12. Patient outcome data were collected to the last follow-up date. This retrospective study was approved by the Ethics Committee in Research of Hospital of Ribeirão Preto, São Paulo, Brazil (HCRP), numbers CAAE 60032122.8.0000.5440 and CAAE 43277221.0.0000.5440, and the Ethics Board of the University of Toronto (Protocol 00043323).

### 2.2. RNA Isolation

RNA extraction was performed on tissues containing tumor-rich areas, which were previously identified and marked by a pathologist (FPS) to represent the highest Gleason pattern. Serial 5 μm formalin-fixed paraffin-embedded (FFPE) tissue was processed at the Ontario Institute for Cancer Research, Toronto, Canada (OICR), using extraction methods described in previous studies [30,31]. 

### 2.3. Transcription Analysis

RNA profiling was performed using both the NanoString PanCancer and the Immune Profiling Panels (NanoString Technologies Inc., Seattle, WA, USA) [32] according to the manufacturer’s instructions. Briefly, RNA profiling using the NanoString methodology relies on digital molecular barcoding and direct hybridization to quantify gene expression levels across multiple genes simultaneously. This methodology has been shown to offer high sensitivity, specificity, and the ability to analyze gene expression patterns from small amounts of RNA, as described previously [33]. The NanoString PanCancer panel comprises 730 genes involved in the cancer progression processes, such as angiogenesis, extracellular matrix remodeling (ECM), EMT, and metastasis. The Immune Profiling Panel comprises 730 immune response genes specifically optimized for immuno-oncology investigative research. There are 130 endogenous genes common between the 2 transcriptional panels, yielding 1200 unique transcripts available for interrogation. Raw expression data from both panels were loaded in nSolver software v4.0 (NanoString Technologies) to perform the quality control (QC) analysis and to build the transcript matrix for downstream analysis. Pearson correlation analysis was performed for the 160 genes in common between the panels and was used to assess reproducibility and identify any potential panel bias. The majority of the 160 genes common to both panels showed a consistent positive correlation between the panels, indicating that gene expression analyses by each panel were reproducible (Supplementary Figure S1). For initial differential expression, we used DESeq2 v1.34.0 with BCR and risk factor as the design factors [34]. We conducted over-representation enrichment analysis (ORA) and Gene Set Enrichment Analysis (GSEA) on the differential expressed genes utilizing the clusterprofiler v4.0 with Kyoto Encyclopedia of Genes and Genomes (KEGG) pathways [35]. Additionally, we categorized the expression levels of *ZEB1* and *SNAI1* into quartiles for each gene. These categorical data allowed us to classify patient gene expressions as either “low” (below Q3) or “high” (above Q3) for *ZEB1* and *SNAI1* [28]. We then used the classification status of *ZEB1* and *SNAI1* as the design factor for the transcriptome analysis as described earlier. For validation purposes, we utilized RNA-seq data from the prostate adenocarcinoma cohort in The Cancer Genome Atlas (PRAD-TCGA, *n* = 420) [36]. We compared the effects of dichotomized expression levels of *ZEB1* and *SNAI1* in this public domain cohort.

### 2.4. Digital Cytometry Analysis

To quantify the immune cell composition in the TME of tumors having a high expression of *ZEB1* and *SNAI1*, we used expression data from TCGA-PRAD [36] analyzed using the digital cytometry resource CIBERSORTx [37]. This algorithm estimates the relative immune abundance in the TME using a “signature matrix” containing validated leukocyte expression data from 22 human hematopoietic cell phenotypes (LM22).

### 2.5. Statistical Analysis

The data processing and downstream analysis for transcriptome data were completed in Rstudio software (R Foundation for Statistical Computing, R v4.1.2 “Bird Hippie”). Multiple unpaired *t*-tests were assessed to calculate the statistical significance using the GraphPad Prism 9.3.0 software for CIBERSORT data. Genes were considered differentially expressed when log2 fold change > 0.5 for the NanoString PanCancer and Immune Profiling Panels, and a more rigorous threshold of >0.58 was used for validation comparisons with the TCGA-PRAD, with p-adjusted (FDR) < 0.05. For the enrichment analysis, we used a cutoff value of 0.05 to consider the ORA of Molecular Signatures Database (MsigDB) Hallmarks. Kaplan–Meier estimates of BCR-free survival were computed using the survival package v3.4.0. Figure S1 illustrates the general workflow of this work (Supplementary Figure S2).

## 3. Results

### 3.1. Identification of DEGs and Pathways Associated with BCR and Clinical Risk

The NanoString PanCancer and the Immune Profiling Panel were developed to cover cancer-related biological functions and features related to adaptative and innate immune response genes. Using both panels, we examined the DEGs (log2 fold change > 0.5) to determine the impact of downstream changes in gene expression on PCa progression through outcome and immune evasion pathways. 

In the first phase of our transcriptomic analysis, we investigated DEGs within the FMRP cohort stratified by CAPRA-S and BCR status. Patients with either a CAPRA-S intermediate or a CAPRA-S high relative risk were grouped together as “High”, while the remaining patients identified as low-risk CAPRA-S, were defined by the “Low” group.

Table 1 and Table 2 summarize the significantly associated DEGs with BCR and CAPRA-S determined using the PanCancer panel and Immune Panel, respectively. Many of the DEGs identified in this analysis have been previously reported as prognostic biomarkers in PCa or have been published as potential markers of immune response in various cancers.

**Figure 1:**
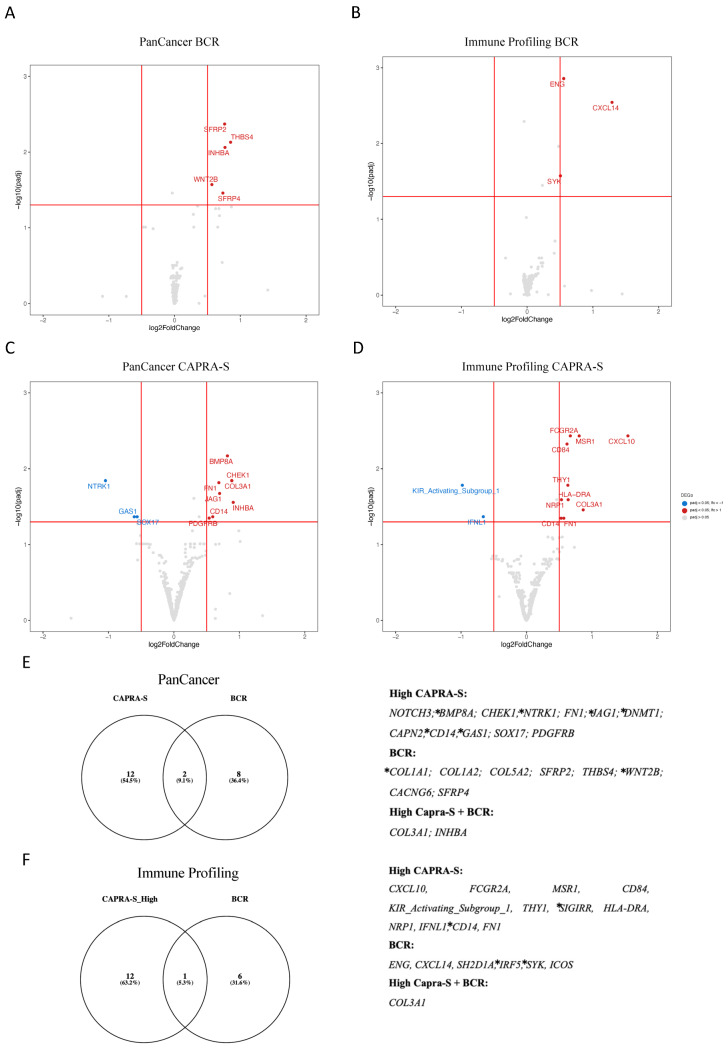
NanoString profile of primary prostate cancer patients. (**A**–**D**) Volcano plots of the DEGs stratified by BCR and CAPRA-S status for both transcriptome panels. (**E**,**F**) Venn diagrams represent the intersection of the DEGs from BCR and CAPRA-S for both transcriptome panels. The list of 39 significantly associated DEGs from both comparisons is displayed on the right side of both panels. Eleven of these DEGs (*COL1A1*, *WNT2B*, *BMP8A*, *NTRK1*, *JAG1*, *DNMT1*, *CD14*, *GAS1*, *SIGIRR*, *IRF5*, and *SYK*) marked with * were identified as target genes for the EMT transcription factor *ZEB1* using the ENCODE transcription factor dataset that has all 8646 target genes of *ZEB1* based on ChIP-seq [67]. Both clinical comparisons used no BCR and low CAPRA-S scores as references from the FMRP cohort. Adjusted *p*-value < 0.05 and a log2 fold change > 0.5. Data were plotted using *ggplot2* and Venny (https://bioinfogp.cnb.csic.es/tools/venny/index.htm, accessed on 5 December 2023).

Volcano plot analysis of the significantly altered DEGs associated with BCR included the overexpression of *SFRP2*, *THBS4*, *INHBA*, *WNT2B*, and *SFRP4* (Figure 1A), as well as *ENG*, *CXCL14*, and *SYK* (Figure 1B) amongst the “Low” CAPRA-S group. Similarly, the “High” CAPRA-S group exhibited a substantial number of DEGs (Figure 1C,D). Association with CAPRA-S showed increased expression of *BMP8A*, *CHEK1*, *FN1*, *COL3A1*, *JAG1*, *CD14*, *INHBA*, and *PDGFRB* (PanCancer) and *FCGR2A*, *CD84*, *MSR1*, *CXCL10*, *THY1*, *HLA-DRA*, *COL3A1*, *NRP1*, *CD14*, and *FN1* (Immune Panel). Reduced expression of *NTRK1*, *GAS1*, and *SOX17* (PanCancer) and *KIR_Activating_Subgroup 1* and *IFNL1* (Immune Panel) was also identified. Amongst the overlapping genes between panels, increased expression of *CD14*, *FN1*, and *COL3A1* were independently detected and associated with high CAPRA-S (Figure 1E,F). 

The last part of our investigation was to identify pathways based on ORA and GSEA analyses of the DEGs identified. Enrichment analysis revealed pathways related to angiogenesis, and TGF-beta, EMT, and UV response were associated with progression and immune response in the FMRP cohort (Supplementary Table S2). Underscoring the importance of the EMT in PCa progression, 11 (28%) of the 39 DEGs (Figure 1F) associated with BCR or CAPRA-S in our cohort were identified as target genes for the EMT transcription factor *ZEB1* [67].

Thus, our initial analyses revealed DEGs associated with immune responses and progression, some of which are regulated by the EMT driver ZEB1. Additionally, genes implicated in the remodeling of the TME, including members of the collagen family (*COL1A*, *COL1A2*, *COL3A1*, *COL5A2*) [38], fibronectin (*FN1*) [52], and *SFRP4* [47], were identified as putative markers of PCa progression. These findings, in conjunction with existing published data (as reviewed in [68]), highlight the potential impact of EMT mechanisms on modulating the immune TME during the progression of PCa.

### 3.2. Downstream Effects of ZEB1 and SNAI1 Expression

The dichotomization of *ZEB1* and *SNAI1* gene expression levels was based on quartile (Q) values, with patients classified as “low” defined as below Q3 compared to those classified as “high” being above Q3. Our objective was to establish a classification system for the DEG patterns linked to the transcriptional activity of these two EMT drivers and the potential impact on downstream pathways involved in PCa progression. DEGs derived from the analysis of both panels classified by *ZEB1* and *SNAI1* expression levels are shown in Supplementary Table S3. 

**Figure 2:**
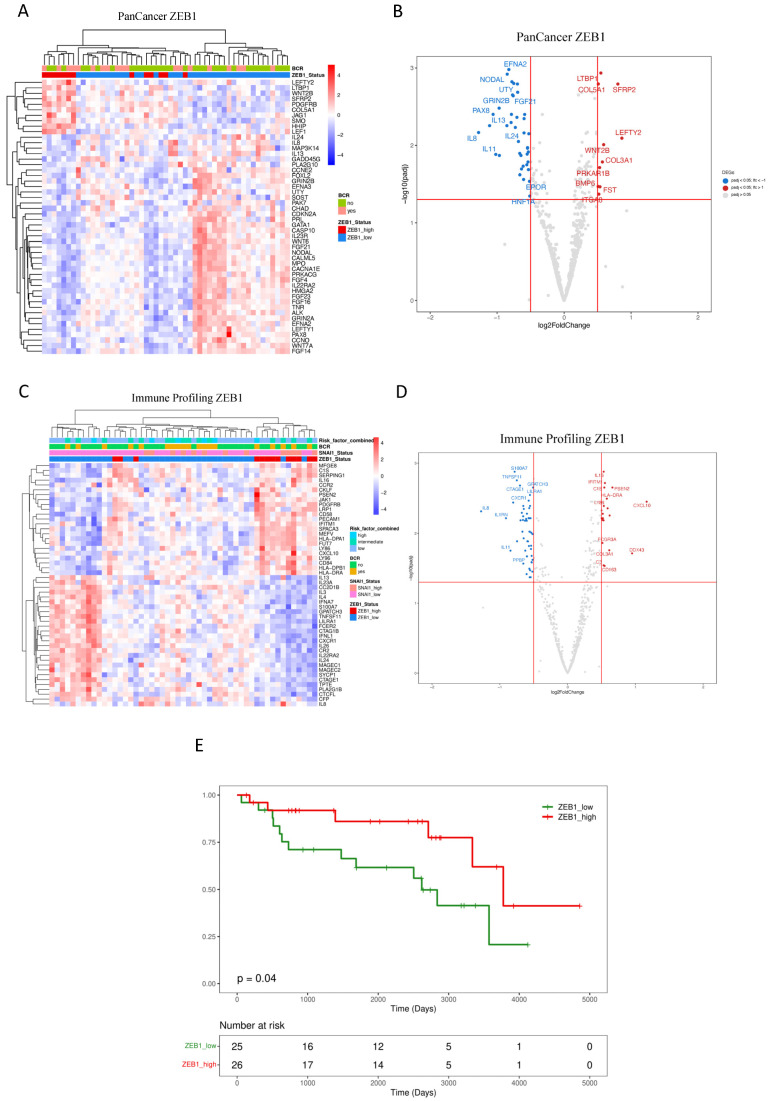
Impact of ZEB1 expression on primary prostate cancer patients. (**A**–**D**) Unsupervised heatmaps and Volcano plots showing the top 50 DEGs for patients expressing high levels of *ZEB1* in the FMRP cohort (*n* = 51). Because the display software is limited by the area available for visualizing the top genes, not all significantly expressed DEGs are depicted in the Volcano plots. (**E**) The Kaplan–Meier plot shows that a low level of *ZEB1* expression is associated with a reduced recurrence-free interval (log-rank test, *p* = 0.04). Adjusted *p*-value < 0.05 and a log2 fold change > 0.5. Data were plotted using *pheatmap* and *ggplot2*.

Using an unsupervised approach, potential relationships amongst samples based on *ZEB1* expression profiles were performed and summarized in Figure 2A,B (PanCancer) and Figure 2C,D (Immune Profiling). A distinct cluster of DEGs associated with high *ZEB1* expression was revealed in the PanCancer panel (Figure 2A), identified by a significantly increased expression of *LEFTY2*, *LTBP1*, *WNT2B*, *SFRP2*, *PDGFRB*, *COL5A1*, *JAG1*, *SMO*, *HHIP*, and *LEF1* (Figure 2B), as well as several under-expressed genes, including *EFNA2*, *NODAL*, *UTY*, *IL8*, *IL13*, *IL24*, and *IL11*. Across the genes in the Immune Panel (Figure 2C), a large cluster of DEGs associated with high *ZEB1* expression was observed, including an increased expression of *CKCLF*, *PSEN2*, *JAK1*, *PDGFRB*, *LRP1*, *CD58*, *PECAM1*, *IFITM1*, *SPACA3*, *MEFV*, *HLA-DPA1*, *FUT7*, *LY86*, *CXCL10*, *LY96*, *CD84*, *HLA-DPB1*, and *HLA-DRA* (Figure 2D). Similarly, an under-expressed cluster of genes included *IL8*, *TNFSF11*, *CXCR1*, and *IL11* (Figure 2D). Pathway analysis of the up- and downregulated DEGs was performed using ORA. *ZEB1* expression was associated with allograft rejection, inflammatory response, interferon-alpha and interferon-gamma response, EMT, IL-2/STAT5, IL-6/JAK/STAT3, WNT/beta-catenin (Supplementary Table S4). 

The elevated *ZEB1* expression demonstrated a statistically significant association with a reduced risk of BCR for our cohort of intermediate-risk PCa, as determined by Kaplan–Meier analysis, (log-rank test *p* = 0.04) (Figure 2E). This observation aligns with previous findings in which reduced *ZEB1* expression was associated with aggressive disease in PCa [69].

**Figure 3:**
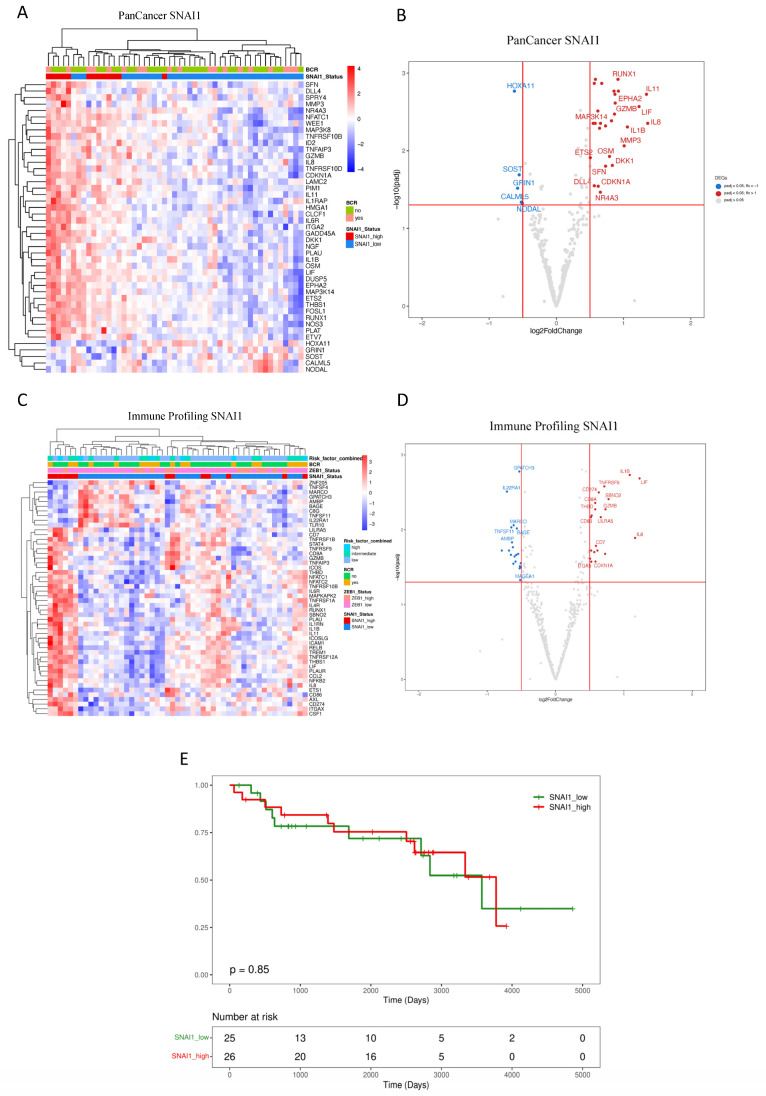
Impact of SNAI1 expression on primary prostate cancer patients. (**A**–**D**) Unsupervised heatmaps and Volcano plots showing the top 50 DEGs for patients expressing high levels of *SNAI1* in the FMRP cohort (*n* = 51). (**E**) The Kaplan–Meier plot shows that the recurrence interval is not affected by levels of *SNAI1* (log-rank test, *p* = 0.85) in the FMRP cohort. Adjusted *p*-value < 0.05 and a log2 fold change > 0.5. Data were plotted using *pheatmap*.

Transcriptome analysis based on the dichotomization of *SNAI1* uncovered several DEGs illustrated in Figure 3A,B (PanCancer) and Figure 3C,D (Immune Profiling). Results from the PanCancer panel demonstrated clustering associated with high *SNAI1* expression, revealing a reduced expression of *HOXA11*, *GRIN1*, *SOST*, *CALML5*, and *NODAL* (Figure 3A,B). Significantly overexpressed genes include *RUNX1*, *ETS2*, *IL1B*, *LIF*, and *IL8*. Similarly, DEGs related to *SNAI1* expression from the Immune Profiling Panel included the overexpression of *CD274* (*PDL1*), *IL1B*, and *TNFRSF9* (Figure 3C,D).

Enrichment analysis of DEGs identified pathways, including TNF-alpha via NF-kB, hypoxia, p53, and PI3K/AKT/mTOR, in addition to those identified in our analysis linked with *ZEB1* expression, including IL-2/STAT5, IL-6/JAK/STAT3, EMT, and interferon-alpha and interferon-gamma response (Table 3). Kaplan–Meier analysis revealed no significant association between *SNAI1* and BCR-free survival (*SNAI1* high vs. low expression, log-rank test, *p* = 0.85) (Figure 3E). 

### 3.3. Impact of ZEB1 and SNAI1 Expression on the Immune TME 

**Figure 4:**
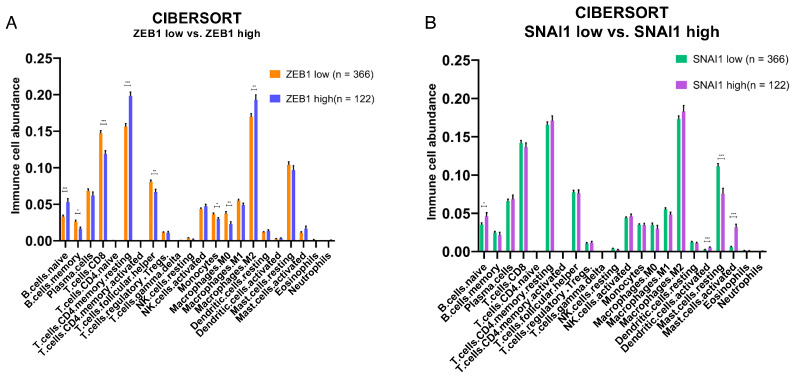
Effects of a high and low expression of EMT transcription factors on the relative abundance of immune cells in the TME of the PRAD-TCGA cohort. Deconvolution-based digital cytometry shows that expression levels of EMT transcription factors influence the relative abundance of immune cell content in the TME. (**A**) The high *ZEB1* group showed an increased abundance of naïve B cells, resting memory CD4+ T cells, and M2 macrophages, and a decreased abundance of memory B cells, CD8 T cells, follicular T helper cells, monocytes, and M0 macrophages. (**B**) *SNAI1* shows an increased abundance of naïve B cells, resting dendritic cells, and activated mast cells, and a decreased abundance of resting mast cells. Results derived from public domain data (TCGA-PRAD). * *p*-value < 0.05; ** *p*-value < 0.01; *** *p*-value < 0.001 by Mann–Whitney test.

To investigate whether tumors expressing high *ZEB1* and *SNAI1* levels affect the variation in immune cell composition in PCa, we investigated the relative abundance of immune cells using TCGA-PRAD public domain transcriptomic data. CIBERSORTx analysis was used to determine the impact of “high” vs. “low” expression levels of *ZEB1* and *SNAI1* on TILs and the immune content of the TME. Our results showed that *ZEB1’s* high expression was associated with an increased abundance of naïve B cells, resting memory CD4+ T cells, and M2 macrophages, and a decreased abundance of memory B cells, CD8 T cells, follicular T helper cells, monocytes, and M0 macrophages (Figure 4A), whilst *SNAI1’s* high expression showed an increased presence of dendritic and B cells (Figure 4B).

The changes in the immune response in the TME associated with the altered expression of *ZEB1* and *SNAI1* suggested that these EMT-related transcription factors may directly or indirectly alter the expression of immune modulatory molecules. For example, we found that the expression of the checkpoint gene *CD274* (*PD-L1*) was associated with *SNAI1’s* high expression in our retrospective cohort analysis (Figure 3C,D). We, therefore, investigated whether the expression of *ZEB1* and *SNAI1* was also associated with changes in the expression of specific immune checkpoints and immune evasion-related markers in the TCGA-PRAD cohort. Our analysis showed that a high expression of the EMT transcription factors *ZEB1* and *SNAI1* is associated with an elevated expression of *CTLA-4*, *PD-L1*, *HAVCR2* (*TIM-3*), *DCR3*, and *IL10*, and *IL10RA* (Supplementary Figures S3 and S4), suggesting a pattern of the upregulation of immunomodulatory genes resulting in an increase in the composition of immune cells of the TME. Also, these findings collectively highlight the role of the TME in shaping the gene expression signature and outcome in PCa.

## 4. Discussion

An important hallmark of cancer is understanding how tumors manage to evade the host immune system [70]. This is a crucial adaptive advantage for survival, maintenance, and the evolution of cancer, especially after the emerging success of different types of immunotherapies. PCa is a tumor considered immunologically cold, that is, a type of tumor that is successful in immune evasion and, consequently, does not respond well to immunotherapy [7,8].

The dynamic and reversible nature of the EMT program impacts not only the tumor cells but also the surrounding ECM by accumulating immune suppressive cells in the TME and upregulating immunomodulatory molecules [22]. In PCa, EMT pathways have already been shown to be strongly related to characteristics of progression and aggressiveness, such as migration, invasion, and increased metastatic potential [14,18]. There is growing evidence suggesting that a partial EMT phenotype, in which cells can simultaneously maintain both epithelial and mesenchymal characteristics, may lead to more aggressive disease than a complete EMT [69]. This observation is consistent with our finding that higher *ZEB1* expression was associated with a reduced risk of BCR.

Proteins in the collagen family play critical roles in diverse cellular processes, including cell adhesion, migration, differentiation, and proliferation. Collagens in the ECM can engage integrins on tumor cells, impede T cell infiltration, interact with CAFs, and facilitate invasion and metastasis [71]. Of the thirty-nine DEGs in our cohort significantly associated with BCR or CAPRA-S, we identified an increased expression of four collagen genes (*COL1A1*, *COL1A2*, *COL3A1*, and *COL5A2*). Of these, *COL3A1* (collagen type III alpha 1) is the most common DEG in our series, and it is an established biomarker of poor outcome in PCa [38,72]. Its expression also appears to promote immune infiltration in a wide variety of different cancers [73]. *COL3A1* interacts with fibronectin (*FN1*), which was also found to be significantly overexpressed. A crucial component of the ECM, FN1, is also intricately associated with collagens and CAFs [52]. Similarly, increased expression of *INHBA* (inhibin β A) was also observed from our results and is associated with enhanced collagen expression, including both *COL3A1* and *COL5A2* [74]. An increased expression of *COL3A1* in PCa activates other pro-tumorigenic genes and pathways, such as the Wnt/beta-catenin [38]. *COL3A1* expression is associated with higher Gleason scores, higher PSA levels, and a higher likelihood of lymph node involvement. Additionally, *COL5A2* expression correlated with increased tumor cell invasion and resistance to androgen deprivation therapy [40]. *SFRP2* was identified as a regulator of the TME through its impact on Wnt signaling and tumor angiogenesis [41,42], while *THBS4* influenced cancer stem cell-like properties in PCa via the PI3K/Akt pathway [43]. Several other DEGs have been previously associated with higher Gleason scores, including *COL1A2* and *INHBA* (subunit of Activin A) [44,45]. *WNT2B* is regulated by long non-coding RNAs (lncRNAs) and has been shown to play a role in influencing the EMT in PCa [46], while *SFRP4* emerged as a predictor of BCR in PCa, and its expression is also linked to the EMT [47]. 

Analysis of the immune-related components identified several DEGs that could be involved in shaping the immune landscape of PCa that were also associated with disease progression defined by CAPRA-S and BCR status (Table 2). The expression of Interferon Regulatory Factor 5 (*IRF5*) was associated with BCR, suggesting that this immune response modulator may also influence prognosis [58]. Similarly, the observation that *THY1* is overexpressed in PCa-associated fibroblasts may also be involved in antigen presentation in the stromal components of the TME of PCa [63]. The identification of *HLA-DRA* in our analysis offers the possibility that dysregulation may affect antigen presentation within the TME and influence the immune response [65]. *NRP* has been reported to be upregulated by androgen deprivation therapy (ADT) in advanced PCa [66], and its expression is thought to lead to increased vascularization and facilitate tumor progression. The co-expression of the immune cytokines *CXCL10* with *CXCR3* has been previously associated with metastatic recurrence [60]. 

It is noteworthy that of our 39 high-risk significantly associated DEGs, *ENG*, *INHBA*, *COL1A1*, *COL3A1*, *COL5A2*, *SFRP4*, *THY1*, and *CXCL14* were also identified as prognostic biomarkers in a recently published transcriptional signature predictive of recurrence [75]. *COL3A1*, *FN1*, and *THBS4* were found to be associated with high infiltration of Tregs in bone metastatic PCa [72]. Similarly, a recent patent identified *COL1A1*, *FN1*, *COL3A1*, *INHBA*, and *SFRP4* as stromal response genes that can be used to test for PCa outcome [76].

Eleven of the thirty-nine significant DEGs were identified as *ZEB1* target genes using the Harmonizome database [67]. Nine of the eleven ZEB1 target genes were associated with high CAPRA-S. The proteins encoded by these 11 DEGs, *COL1A1*, *WNT2B*, *IRF5*, *SYK*, *BMP8A*, *NTRK1*, *JAG1*, *DNMT1*, *CD14*, *GAS1*, and *SIGIRR*, collectively present a set of functional properties that may be integral in limiting the immune response within the TME of PCa when an EMT transcriptional program is regulated by *ZEB1*. For example, collagen production by COL1A1 may physically impede immune cell infiltration [77], while WNT2B signaling is associated with immunosuppression [46]. IRF5, involved in immune regulation, and SYK, have been implicated in immune cell functions [59]. Additionally, BMP8A, NTRK1, JAG1, and GAS1 each bring unique contributions that can influence the immunosuppressive characteristics of the TME [49,51,53,54]. Furthermore, DNMT1 and CD14, through epigenetic regulation and immune cell activation, respectively, contribute to the overall immune evasion [78]. Lastly, SIGIRR’s role as a negative regulator of Toll-like receptor signaling suggests its potential involvement in immune suppression [64].

Validation using the PRAD TCGA public cohort showed that both EMT drivers *ZEB1* and *SNAI1* are associated with the expression of the immunological evasion markers *CTLA-4*, *PD-L1*, *TIM3*, *DCR3*, and *IL10*. The expression of these markers leads not only to an inactivation of T cells but also to a generalized immune suppression in the TME [79]. Checkpoint proteins like CTLA-4 and PD-L1 inhibit T cell activation by delivering inhibitory signals to T cells upon engagement with their respective ligands. This inhibition prevents the full activation of T cells, leading to a state of quiescence where T cells remain inactive and are unable to mount an effective immune response against tumors [80]. 

Analysis of the relative abundance of immune cells in the TME of the PRAD TCGA cohort showed that *ZEB1* expression was associated with an increase in M2 polarization macrophages, which are known to be involved in the suppression of immunological activity [81]. Decreases in memory B cells, CD8+ T cells, follicular T helper cells, monocytes, and M0 macrophages further support *ZEB1* expression and may influence anti-tumor immunity in the TME and recruitment of TILs. In contrast, digital cytometric analysis of the effects of high *SNAI1* expression on the TME correlated with increased abundance of naïve B cells, resting dendritic cells, and activated mast cells, while showing decreased levels of resting mast cells. Interestingly, mast cell infiltration in PCa has been associated with chemotherapy resistance through the activation of p38/p53/p21 signaling [82]. Collectively, these data suggest that downstream changes activated by EMT transcription factors not only influence the aggressive behavior of tumors but also lead to changes in the immune activity of the TME. 

## 5. Conclusions

In summary, these data suggest that the differential expression of collagen genes, such as *COL3A1* and various immune response genes observed in our study, are part of the EMT program, leading to cellular alterations that impact immune cell functions in the microenvironment of PCa. Collagen-related signals can modulate T cell activation, proliferation, and cytokine production. Moreover, the density and organization of collagen fibers could affect the spatial distribution and activation levels of immune cells within the tumor, influencing their ability to recognize and eliminate cancer cells. Understanding the interplay between the spatial effects of collagen and immune cells in the TME has therapeutic implications. This study has some limitations. The CAPRA-S score, which was used to classify the groups according to tumor progression relies on pathological factors, like the Gleason score and tumor stage. While these are important, they may not fully capture the complexity of prostate cancer biology and its interaction with the host environment. Furthermore, our sample size of 51 cases, while limited, was aimed at providing pilot data to establish a connection between the EMT and the immune TME in prostate cancer, thereby providing a basis for future clinical investigations with larger cohorts. This study suggests that future treatment strategies aimed at modulating the EMT [14,83] may enhance immune cell infiltration toward an anti-tumorigenic phenotype, which could be beneficial for countering immunotherapy resistance in a cold tumor, such as PCa.

## Figures and Tables

**Table 1:** Ranked list of the DEGs associated with BCR and CAPRA-S based on the PanCancer Panel using the FMRP cohort. The roles of each of the top-ranking DEGs in the cancer progression and PCa literature are shown with specific citations (if available). Adjusted *p*-value < 0.05 and a log2 fold change > 0.5.

	Gene	Log2 FC	padj	Protein	Role in Progression and Biology of PCa	Citations
BCR	*COL1A1*	0.876	>0.001	collagen type I alpha 1 chain	Collagens contribute to the ECM, which are the major structural components of the TME. *COL1A1*, *COL1A2*, and *COL3A1* expression in CAFs have been associated with the EMT. *COL1A1* expression is upregulated in PCa stromal cells and was associated with a worse prognosis in PCa.	[38,39]
	*COL3A1*	0.879	>0.001	collagen type III alpha 1 chain	*COL3A1* interacts with fibronectin. Increased expression of *COL3A1* in PCa activates other pro-tumorigenic genes and pathways, such as the Wnt/beta-catenin. *COL3A1* expression is associated with higher Gleason scores, higher PSA levels, and a higher likelihood of lymph node involvement.	[38]
	*COL1A2*	0.596	>0.001	collagen type I alpha 2 chain	*COL1A2* expression is associated with higher Gleason scores.	[40]
	*COL5A2*	0.479	>0.001	collagen type V alpha 2 chain	*COL5A2* expression has been associated with increased tumor cell invasion and resistance to androgen deprivation therapy.	[40]
	*SFRP2*	0.761	0.004	secreted frizzled-related protein 2	*SFRP2* affects TME by regulating Wnt signaling and influencing tumor angiogenesis.	[41,42]
	*THBS4*	0.852	0.007	thrombospondin 4	*THBS4* affects cancer stem cell-like properties in PCa by its regulation of the PI3K/Akt pathway.	[43]
	*INHBA*	0.766	0.008	inhibin beta A subunit	*INHBA* (Activin A) activates NF-κB and is associated with a higher Gleason score PCa.	[44,45]
	*WNT2B*	0.567	0.02	Wnt family member 2B	*WNT2B* is regulated by lncRNAs to influence the EMT in PCa.	[46]
	*SFRP4*	0.7349	0.03	secreted frizzled-related protein 4	*SFRP4* predicts BCR in PCa and is associated with the EMT.	[47]
CAPRA-S	*NOTCH3*	0.6882	>0.001	notch 3	*NOTCH1-4* expression was associated with disease progression, prognosis, and immune cell infiltration.	[48]
	*BMP8A*	0.8167	0.006	bone morphogenetic protein 8a	BMPs are members of the TGF-beta family and are thought to be involved in PCa bone metastasis.	[49]
	*CHEK1*	0.8851	0.01	checkpoint kinase 1	*CHEK1* (*CHK1*) is associated with DNA damage response and AR signaling.	[50]
	*COL3A1*	0.8822	0.01	collagen type III alpha 1 chain	see above	
	*NTRK1*	−1.045	0.01	neurotrophic receptor tyrosine kinase 1	*NTRK1* downregulation is associated with reduced TILs in the TME of PCa and poor prognosis.	[51]
	*FN1*	0.686	0.01	fibronectin 1	*FN1* is a key component of the ECM, and the TME is associated with collagens and CAFs.	[52]
	*JAG1*	0.697	0.02	jagged 1	*JAG1* upregulation results in increased inflammatory foci in the TME of tumors in *Pten*-deficient mice.	[53]
	*INHBA*	0.905	0.02	inhibin beta A subunit	See above.	
	*CD14*	0.592	0.04	CD14 molecule	*CD14* is mostly expressed in macrophages.	--
	*GAS1*	−0.606	0.04	growth arrest-specific 1	*GAS1RR* (an immune-related enhancer RNA) represses *GAS1* and is associated with BR-free survival in PCa.	[54]
	*SOX17*	−0.561	0.04	SRY-box 17	*SOX17* and Notch’s axis associated with enzalutamide resistance in CRPC models.	[55]

**Table 2:** Ranked list of the DEGs associated with BCR and CAPRA-S based on the Immune Profiling Panel using the FMRP. The roles of each of the top-ranking DEGs in immune oncology and the PCa literature are shown with specific citations (if available). Adjusted *p*-value < 0.05 and a log2 fold change > 0.5.

	Gene	Log2 FC	padj	Protein	Role in Immune Oncology and PCa	Citations
BCR	*COL3A1*	1.027	>0.001	collagen type III alpha 1 chain	See Table 1.	
	*ENG*	0.556	0.001	endoglin	Endoglin (sCD105) in plasma associated with aggressive PCa.	[56]
	*CXCL14*	1.291	0.002	C-X-C motif chemokine ligand 14	*CXAL14* expression associated with outcome in PCa.	[57]
	*SH2D1A*	−0.045	0.005	SH2 domain containing 1A	Stimulation factor for T and B cells.	--
	*IRF5*	0.478	0.01	interferon regulatory factor 5	IRF5 expression used for BCR prediction in PCa.	[58]
	*SYK*	0.506	0.02	spleen associated tyrosine kinase	Associated with metastatic PCa.	[59]
	*ICOS*	0.231	0.03	inducible T cell costimulator	ICOS + Treg cells exert immunosuppressive effects.	--
CAPRA-S	*CXCL10*	1.549	0.003	C-X-C motif chemokine ligand 10	CXCL10 co-expression with *CXCR3* is a predictor of metastatic recurrence.	[60]
	*FCGR2A*	0.669	0.003	Fc fragment of IgG receptor IIa	Expressed in macrophages, neutrophils, and other immune cells.	--
	*MSR1*	0.804	0.003	macrophage scavenger receptor 1	Helpful as an additional diagnostic biomarker for PCa.	[61]
	*CD84*	0.618	0.004	CD84 molecule	Expressed in numerous immune cell types.	--
	*KIR Activating* *Subgroup 1*	−0.98	0.01	killer cell immunoglobulin-like receptors	KIRs expressed in NK and T cells.	[62]
	*THY1*	0.631	0.01	Thy-1 cell surface antigen	*THY1* overexpressed in PCa-associated fibroblasts.	[63]
	*SIGIRR*	0.459	0.02	single Ig and TIR domain containing	*TLR4* and IL-1R-mediated NF-kB activation associated with BCR.	[64]
	*HLA-DRA*	0.636	0.02	major histocompatibility complex, class II, DR alpha	Antigen presentation in the TME.	[65]
	*NRP1*	0.534	0.02	neuropilin 1	Androgen-repressed gene upregulated by ADT in advanced PCa.	[66]
	*COL3A1*	0.865	0.03	collagen type III alpha 1 chain	See Table 1.	
	*IFNL1*	−0.66	0.04	interferon lambda 1	Interferon lambda 1 is involved in antiviral immune defense.	--
	*CD14*	0.528	0.04	CD14 molecule	*CD14* is mostly expressed in macrophages.	--
	*FN1*	0.570	0.04	fibronectin 1	See Table 1.	

**Table 3:** Enrichment analysis from DEGs associated with *SNAI1* High expression in the HC-FMRP cohort. List of ORA enriched pathways using MSigDb Hallmark’s terms for DEGs associated with high expression of *SNAI1*. The analysis used patients with low expression of *SNAI1* as a reference.

Term	Adjusted *p*-Value	Genes
Immune Profiling		
TNF-alpha Signaling via NF-kB	>0.001	*EGR2*; *CDKN1A*; *CSF1*; *CD80*; *TNFRSF9*; *LIF*; *PLAUR*; *TNFAIP3*; *NFKB1*; *ICAM1*; *NFKB2*; *RELB*; *NFKBIA*; *BCL6*; *PLAU*; *IL1B*; *REL*; *CCL2*; *ICOSLG*; *CD44*
Inflammatory Response	>0.001	*CDKN1A*; *IL4R*; *CSF1*; *TNFRSF9*; *IL10RA*; *LIF*; *PLAUR*; *ICAM4*; *TNFRSF1B*; *NFKB1*; *ICAM1*; *NFKBIA*; *MARCO*; *IRAK2*; *AXL*; *IL1B*; *IRF7*; *CCL2*; *ITGA5*; *ICOSLG*
Allograft Rejection	>0.001	*CD86*; *CCR1*; *IL11*; *IL4R*; *CSF1*; *CD80*; *LIF*; *GZMB*; *ETS1*; *ICAM1*; *NCR1*; *CD8A*; *IL1B*; *IL9*; *CD7*; *IRF7*; *STAT4*; *CCL2*; *IL12A*; *ICOSLG*
Interferon-Gamma Response	>0.001	*CD86*; *CD274*; *CDKN1A*; *IL4R*; *VCAM1*; *IL10RA*; *TNFAIP3*; *NFKB1*; *ICAM1*; *NFKBIA*; *IRF7*; *STAT4*; *TXNIP*; *CCL2*
IL-2/STAT5 Signaling	>0.001	*CD86*; *IL4R*; *CSF1*; *TNFRSF9*; *IL10RA*; *LIF*; *ITGAE*; *TNFRSF1B*; *MAPKAPK2*; *TNFSF11*; *CTLA4*; *ICOS*; *CD44*
IL-6/JAK/STAT3 Signaling	>0.001	*CCR1*; *IL4R*; *CSF1*; *TNFRSF12A*; *IL1B*; *TNFRSF1B*; *CD44*; *TNFRSF1A*
KRAS Signaling Up	>0.001	*PLAU*; *ITGA2*; *IL1B*; *IL10RA*; *LIF*; *PLAUR*; *TNFAIP3*; *TNFRSF1B*; *ETS1*
Coagulation	>0.001	*THBD*; *C8G*; *PLAU*; *ITGA2*; *C8A*; *THBS1*; *SH2B2*
Epithelial–Mesenchymal Transition	>0.001	*VCAM1*; *TNFRSF12A*; *ITGA2*; *PLAUR*; *TNFAIP3*; *ITGA5*; *THBS1*; *CD44*
Apical Junction	>0.001	*CD86*; *CD274*; *VCAM1*; *ITGA2*; *ICAM4*; *CD34*; *ICAM1*
Apoptosis	0.005	*CDKN1A*; *TNFRSF12A*; *IL1B*; *TXNIP*; *CD44*
Interferon-Alpha Response	0.005	*IL4R*; *CSF1*; *IRF7*; *TXNIP*
PanCancer		
TNF-alpha Signaling via NF-kB	>0.001	*DUSP5*; *CDKN1A*; *GADD45A*; *LIF*; *TNFAIP3*; *ETS2*; *FOSL1*; *NR4A3*; *PLAU*; *CLCF1*; *ID2*; *IL1B*; *MAP3K8*
Epithelial–Mesenchymal Transition	>0.001	*GADD45A*; *ITGA2*; *ID2*; *MMP3*; *TNFAIP3*; *LAMC2*; *THBS1*; *DKK1*
KRAS Signaling Up	>0.001	*PLAU*; *ITGA2*; *ID2*; *IL1B*; *LIF*; *TNFAIP3*; *PLAT*; *NGF*
Coagulation	>0.001	*PLAU*; *ITGA2*; *MMP3*; *PLAT*; *THBS1*
Apoptosis	>0.001	*CDKN1A*; *WEE1*; *GADD45A*; *IL1B*; *PLAT*
Complement	>0.001	*DUSP5*; *PIM1*; *TNFAIP3*; *GZMB*; *PLAT*
p53 Pathway	>0.001	*CDKN1A*; *GADD45A*; *LIF*; *SFN*; *EPHA2*
IL-6/JAK/STAT3 Signaling	0.002	*IL1B*; *PIM1*; *MAP3K8*
IL-2/STAT5 Signaling	0.002	*SPRY4*; *PIM1*; *LIF*; *MAP3K8*
Inflammatory Response	0.002	*CDKN1A*; *IL1B*; *LIF*; *OSM*
Allograft Rejection	0.002	*IL11*; *IL1B*; *LIF*; *GZMB*
PI3K/AKT/mTOR Signaling	0.003	*CDKN1A*; *SFN*; *NGF*
TGF-beta Signaling	0.01	*ID2*; *THBS1*
Hypoxia	0.01	*CDKN1A*; *PIM1*; *TNFAIP3*
Estrogen Response Late	0.01	*ID2*; *LAMC2*; *SFN*
Interferon-Gamma Response	0.01	*CDKN1A*; *PIM1*; *TNFAIP3*
E2F Targets	0.01	*CDKN1A*; *WEE1*; *HMGA1*
Xenobiotic Metabolism	0.01	*ID2*; *ETS2*; *EPHA2*

## Data Availability

The datasets presented in this study can be found in online repositories. The names of the repository/repositories and accession number(s) can be found in the article/Supplementary Materials.

## Supplementary Materials

The following supporting information can be downloaded at https://www.mdpi.com/article/10.3390/cancers16081480/s1, Table S1: Prostate cancer tissue samples and validation cohort. BCR, biochemical recurrence; CAPRA-S, Cancer of the Prostate Risk Assessment Score; pGS, pathologic Gleason score; ISUP, International Society of Urological Pathology Score; TNM, Classification of Malignant Tumors. * For validation comparisons, we used RNA-seq data from the prostate adenocarcinoma cohort from The Cancer Genome Atlas (PRAD-TCGA, *n* = 420) [36]. Table S2: Enrichment analysis from the DEGs associated with BCR and CAPRA-S in the FMRP cohort. List of ORA enriched analysis using MSigDb Hallmark’s terms for DEGs associated with BCR and CAPRA-S using both the Immune Profile and PanCancer panels. Both comparisons used no BCR and low CAPRA-S scores as references from the FMRP cohort. Table S3: DEGs based on *ZEB1* and *SNAI1*. List of DEGs from Immune Profiling and PanCaner panels for patients classified according to *ZEB1* and *SNAI1* expression. The genes were considered DE when log2 FC > 0.5 and p-adjusted (FDR) < 0.05. Table S4: Enrichment analysis from DEGs associated with a high *ZEB1* expression in the FMRP cohort. List of ORA-enriched pathways using MSigDb Hallmark’s terms for DEGs associated with a high expression of *ZEB1*. The analysis used patients with a low expression of *ZEB1* as a reference. Figure S1: Correlation plots of common genes between Immune Profiling and PanCancer panels. Pearson correlation was used in normalized expression levels of each gene. The heatmap shows the Pearson correlation coefficient. Non-significant results are displayed in white and significant correlations are colored (*p* < 0.01). Figure S2: Summary workflow. We conducted NanoString panel profiling using RNA extracted from formalin-fixed paraffin-embedded (FFPE) tissues from the Faculty of Medicine of Ribeirao Preto (FMRP) cohort (*n* = 51). DEGs and pathway and downstream analyses of *ZEB1* and *SNAI1* were performed using our in-house pipeline (see methods). Figure S3: Effects of a high and low *ZEB1* expression on the relative expression of checkpoint genes. Analysis of the expression of known immunomodulatory markers shows an increased relative expression of *CTLA-4*, *PD-L1*, *HAVCR2* (*TIM-3*), *IDO1*, *DCR3*, *IL10*, and *IL10RA* in the high *ZEB1* group (*n* = 366) compared to the low group (*n* = 122) in the TCGA cohort. * *p* < 0.05, Mann–Whitney test. Figure S4: Effects of a high and low *SNAI1* expression on the relative expression of checkpoint genes. Analysis of the expression of known immunomodulatory markers shows an increased relative expression of *CTLA-4*, *PD-L1*, *HAVCR2* (*TIM-3*), *IDO1*, *DCR3*, *IL10*, and *IL10RA* in the high *SNAI1* group (*n* = 366) compared to the low group (*n* = 122) in the TCGA cohort. * *p* < 0.05, Mann–Whitney test.
