# Supplementary material for: Investigating the Role of SNAI1 and ZEB1 Expression in Prostate Cancer Progression and Immune Modulation of the Tumor Microenvironment
Source: Cancers (Basel). 2024 Apr 12;16(8):1480. doi: 10.3390/cancers16081480 (PMC11048607; doi:10.3390/cancers16081480)
Supplement: Supplementary file 1 [file cancers-16-01480-s001.zip › Supplementary Figure S3.pdf]

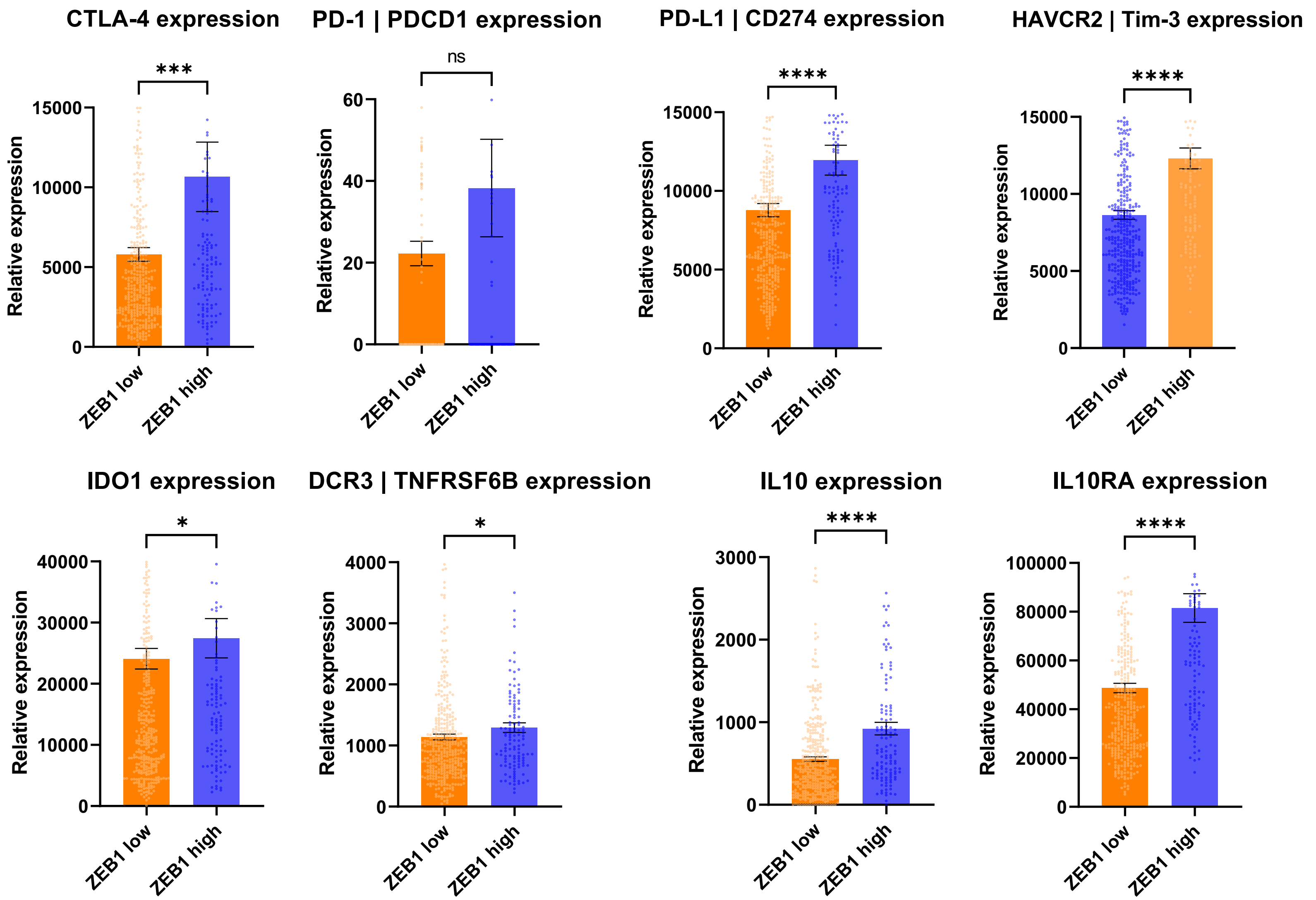

Supplementary Figure S3

Effects of high and low ZEB1 expression on relative expression of checkpoint genes - Analysis of expression of known immunomodulatory markers shows increased relative expression of *CTLA-4, PD-L1, HAVCR2 (TIM-3), IDO1, DCR3, IL10 and IL10RA* in the ZEB1 high group (n =366) compared to low group (n =122) in the TCGA cohort. *P<0.05, Mann-Whitney test.
