# Supplementary material for: Investigating the Role of SNAI1 and ZEB1 Expression in Prostate Cancer Progression and Immune Modulation of the Tumor Microenvironment
Source: Cancers (Basel). 2024 Apr 12;16(8):1480. doi: 10.3390/cancers16081480 (PMC11048607; doi:10.3390/cancers16081480)
Supplement: Supplementary file 1 [file cancers-16-01480-s001.zip › Supplementary Table S1.pdf]

Variable	FMRP n	TCGA• n
**BCR**		
no	32	363
yes	19	57
NA	-	68
**Capra-S**		
low CAPRA-S	20	184
Intermediate CAPRA-S	21	196
High CAPRA-S	10	108
**pGS**		
5	-	-
6	8	44
7	37	242
8	4	62
9	2	136
10	-	3
NA	-	1
**ISUP**		
1	8	-
2	24	-
3	13	-
4	2	-
5	4	-
**TNM**		
pT1c	-	-
pT2	2	-
pT2a	2	13
pT2b	3	10
pT2c	30	162
pT3a	6	155
pT3b	7	131
T4	-	11
NA	-	6
**pre-surgical PSA(ng/ml)**		
0 to 6	16	382
6.01 to 10	12	6
10.01 to 20	19	11
>20	3	4
NA	1	85
**Surgical Margin**		
0	37	309
1	14	150
**Extraprostatic Invasion**		
0	39	185
1	12	297
NA	-	6
**Vesicular Invasion**		
0	50	58
1	1	19
NA	-	411
**Average**		
**Age at surgery**		
66		61

Supplementary Table S1.
