# Supplementary material for: Investigating the Role of SNAI1 and ZEB1 Expression in Prostate Cancer Progression and Immune Modulation of the Tumor Microenvironment
Source: Cancers (Basel). 2024 Apr 12;16(8):1480. doi: 10.3390/cancers16081480 (PMC11048607; doi:10.3390/cancers16081480)
Supplement: Supplementary file 1 [file cancers-16-01480-s001.zip › Supplementary Table S2.pdf]

| | Term | Adjusted P-value | Genes |
|---|---|---|---|
| **PanCancer** | | | |
| **BCR** | Epithelial Mesenchymal Transition | >0.001 | *COL1A1; SFRP4; COL3A1; COL1A2; COL5A2; INHBA* |
| | UV Response Dn | >0.001 | *COL1A1; COL3A1; COL1A2; COL5A2* |
| | Angiogenesis | 0.0003 | *COL3A1; COL5A2* |
| | Myogenesis | 0.008 | *COL1A1; COL3A1* |
| **Immune Profiling** | | | |
| **BCR** | Angiogenesis | 0.06 | *COL3A1* |
| | TGF-beta Signaling | 0.06 | *ENG* |
| | UV Response Dn | 0.06 | *COL3A1* |
| | IL-2/STAT5 Signaling | 0.06 | *ICOS* |
| | Myogenesis | 0.06 | *COL3A1* |
| | KRAS Signaling Up | 0.06 | *ENG* |
| | Interferon Gamma Response | 0.06 | *IRF5* |
| | Apical Junction | 0.06 | *SYK* |
| | Estrogen Response Late | 0.06 | *CXCL14* |
| | Epithelial Mesenchymal Transition | 0.06 | *COL3A1* |
| **CAPRA-S** | Angiogenesis | 0.06 | *NRP1; COL3A1* |
| | Hedgehog Signaling | 0.001 | *NRP1; THY1* |
| | Epithelial Mesenchymal Transition | 0.001 | *COL3A1; FN1; THY1* |
| | Inflammatory Response | 0.001 | *MSR1; CXCL10; CD14* |
| | IL-6/JAK/STAT3 Signaling | 0.005 | *CXCL10; CD14* |
| | UV Response Dn | 0.01 | *NRP1; COL3A1* |
| | IL-2/STAT5 Signaling | 0.01 | *NRP1; CXCL10* |
| | Allograft Rejection | 0.01 | *HLA-DRA; THY1* |
| | KRAS Signaling Up | 0.01 | *NRP1; CXCL10* |

**Supplementary Table S2**
