# Supplementary material for: Investigating the Role of SNAI1 and ZEB1 Expression in Prostate Cancer Progression and Immune Modulation of the Tumor Microenvironment
Source: Cancers (Basel). 2024 Apr 12;16(8):1480. doi: 10.3390/cancers16081480 (PMC11048607; doi:10.3390/cancers16081480)
Supplement: Supplementary file 1 [file cancers-16-01480-s001.zip › Supplementary Table S4.pdf]

Term	Adjusted P-value	Genes
**Immune profiling**		
Allograft Rejection	>0.001	*ITGB2; CFP; ETS1; CD3D; CTSS; ICAM1; CCL7; IL12A; MAP3K7; ELANE; CCR2; IL10; IL11; TGFB2; IL13; LY86; LIF; IL18; IL16; HLA-G; IL4; NCR1; TLR1; PTPRC; CD8B; IL1B; HLA-DRA; IRF8; KLRD1; CD47*
Inflammatory Response	>0.001	*IL10; MSR1; IFITM1; IL10RA; LIF; IL18; PLAUR; OSM; CYBB; ICAM4; MEFV; ICAM1; TLR1; CXCL10; IL1A; CCL7; IRF1; IL1B; C3AR1; CMKLR1*
KRAS Signaling Up	>0.001	*CSF2; IL10RA; ITGB2; LIF; PLAUR; LY96; PPBP; ETS1; CTSS; CXCL10; IL1B; C3AR1; PECAM1; IRF8; CMKLR1*
TNF-alpha Signaling via NF-kB	>0.001	*CSF2; SERPINB2; LIF; IL18; PLAUR; CXCL3; TANK; ICAM1; NFKB2; CXCL10; IL1A; IL23A; IRF1; IL1B*
Interferon Gamma Response	>0.001	*IFITM2; C1S; C1R; IL10RA; HLA-G; ICAM1; CXCL10; CCL7; CASP3; IRF1; CASP1; SERPING1; IRF8; CMKLR1*
Complement	>0.001	*CR2; SERPINB2; LRP1; C1S; C1R; FN1; PLAUR; CTSS; C3; CTSL; CASP3; IRF1; CASP1; SERPING1*
Coagulation	>0.001	*C3; C8G; SERPINB2; C1S; LRP1; C1R; FN1; PECAM1; SERPING1; MASP2*
IL-6/JAK/STAT3 Signaling	>0.001	*CXCL10; CSF2; ITGA4; CCL7; IL1B; IRF1; PDGFC; CXCL3*
Apoptosis	>0.001	*PDGFRB; IL1A; TGFB2; CASP3; IL1B; IRF1; PSEN2; CASP1; IL18; BAX*
Interferon Alpha Response	>0.001	*CXCL10; IFITM1; IFITM2; C1S; IRF1; CASP1; CD47*
IL-2/STAT5 Signaling	>0.001	*IL10; CXCL10; CSF2; CASP3; IL10RA; IL13; LIF; TNFSF11; IRF8*
Epithelial Mesenchymal Transition	0.006	*PDGFRB; ITGB1; COL3A1; CXCL12; LRP1; FN1; PLAUR*
Apical Junction	0.025	*ITGB1; MAPK11; PTPRC; PECAM1 ;ICAM4; ICAM1*
**PanCancer**		
Wnt-beta Catenin Signaling	0.0004	*DKK4; WNT6; JAG1; LEF1*
Epithelial Mesenchymal Transition	0.06	*PDGFRB; COL3A1; COL5A1; LAMA1*
Angiogenesis	0.064	*COL3A1; JAG1*
UV Damage Response	0.09	*PDGFRB; COL3A1; LTBP1*

Supplementary Table S4
