# Supplementary figures and images for: Investigating the Role of SNAI1 and ZEB1 Expression in Prostate Cancer Progression and Immune Modulation of the Tumor Microenvironment
Source: Cancers (Basel). 2024 Apr 12;16(8):1480. doi: 10.3390/cancers16081480 (PMC11048607; doi:10.3390/cancers16081480)

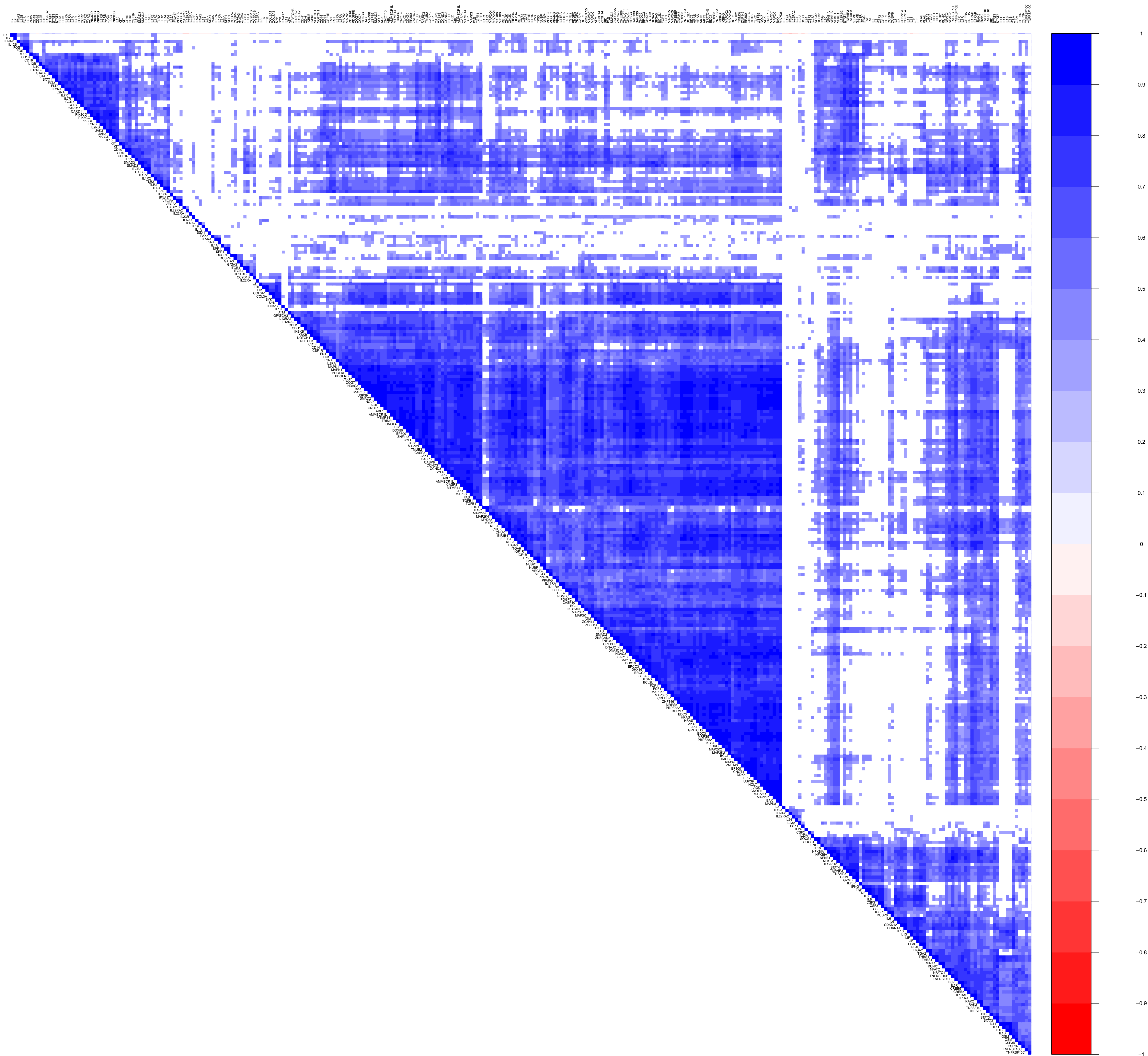

Supplement: Supplementary file 1 [file cancers-16-01480-s001.zip › Supplementary Figure S1.pdf]

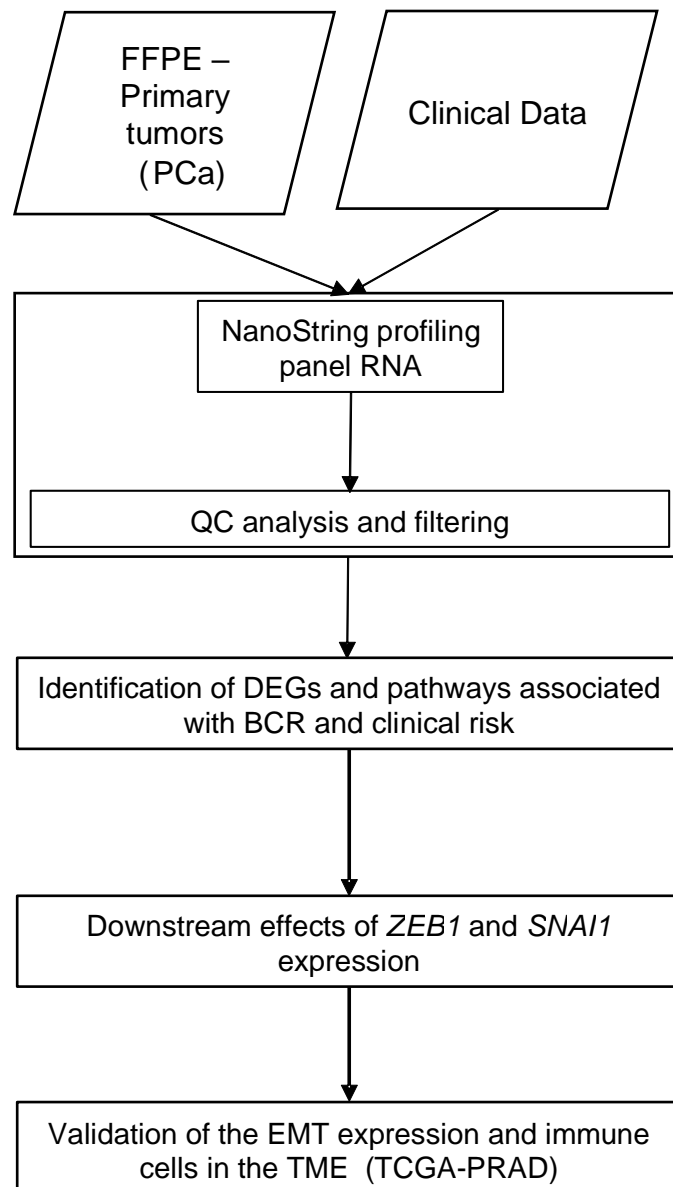

**Supplementary Figure S2**

Supplement: Supplementary file 1 [file cancers-16-01480-s001.zip › Supplementary Figure S2.pdf]
